# Joint Preservation Surgery Using Supramalleolar Osteotomy Combined with Posterior Tibial Tendon Release and Lateral Ligament Augmentation in Advanced Varus Ankle Arthritis

**DOI:** 10.3390/jcm13164803

**Published:** 2024-08-15

**Authors:** Chul Hyun Park, Jeong-Jin Park, In-Ha Woo

**Affiliations:** 1 Department of Orthopaedic Surgery, College of Medicine, Yeungnam University, Daegu 42415, Republic of Korea; 2 Korea Armed Forces Athletic Corps, Mungyeong 36931, Republic of Korea; wjdwls3912@naver.com; 3 ROKA 7th Artillery Brigader, Namyangju 12284, Republic of Korea; buonggiorno39@gmail.com

**Keywords:** ankle, arthritis, tendon, lengthening

## Abstract

**Background**: Recent studies utilizing weight-bearing computed tomography have identified abnormal internal rotation of the talus in advanced varus ankle arthritis (VAA) with a large talar tilt (TT), influenced by the posterior tibial tendon (PTT). This study aimed to evaluate the clinical and radiographic results of supramalleolar osteotomy (SMO) combined with PTT release and lateral ligament augmentation for VAA with a large TT. **Methods**: From January 2015 to September 2018, 15 patients with VAA and a large TT (greater than 5°) underwent SMO combined with PTT release. Clinical results, including visual analog scale (VAS) for pain, American Orthopedic Foot and Ankle Society (AOFAS) ankle-hindfoot score, and ankle osteoarthritis scale (AOS), were assessed. Radiographic results were assessed with various parameters, including medial distal tibial angle (MDTA), anterior distal tibial angle (ADTA), talar tilt (TT), talus center migration (TCM), Meary angle, hindfoot alignment angle (HAA), and hindfoot moment arm (HMA) on foot and ankle weight-bearing radiographs. Clinical and radiographic results were evaluated preoperatively and at the last follow-up. **Results**: VAS, AOFAS ankle-hindfoot score, and AOS improved significantly from 7.5, 54.4, and 72.6 preoperatively to 3.1, 82.5, and 34.5 postoperatively, respectively. All radiographic parameters exhibited significant changes postoperatively, with the exception of the Meary angle, which demonstrated no significant change. Four patients exhibited improvement in radiographic stage postoperatively; however, average radiographic stage did not significantly improve postoperatively in all patients. One patient progressed to end-stage arthritis postoperatively, necessitating additional ankle arthrodesis. **Conclusions**: In conclusion, lengthening and lateral ligament augmentation combined with bony realignment procedures may be a reasonable option for treating VAA with a large TT greater that 5°.

## 1. Introduction

Varus ankle arthritis (VAA) with a large talar tilt (TT) represents a challenging condition to preserve the joint with surgical treatment. Historically, surgical treatment for VAA has focused on correcting the coronal plane in order to redistribute the weight-bearing load to the unaffected areas [1,2,3,4,5,6,7]. Correction of the coronal plane using supramalleolar osteotomy (SMO) and calcaneal osteotomy (CO) has demonstrated favorable outcomes for VAA [2,5,7,8]. However, these methods have proven to be ineffective for VAA with a large TT [5,8].

A recent study using weight-bearing computed tomography (CT) identified that the talus frequently exhibits abnormal internal rotation (IR) in advanced VAA with a large TT [9]. The abnormal IR of the talus is influenced by the posterior tibial tendon (PTT), as it is the primary invertor of the ankle joint [9]. Furthermore, as the ankle arthritis progresses, the medial soft tissues, including ligaments and tendons, generally become tighter. In this condition, the PTT becomes much tighter following the opening wedge SMO due to the lengthening of the medial tibia and the limited excursion of the PTT [10]. Therefore, it can be postulated that medial soft tissue release would be necessary for joint preservation in VAA with a large TT.

A recent study reported that PTT transfer combined with SMO was effective in correcting VAA with a large TT by converting a deforming force into a reduction force for abnormal IR of the talus [11]. However, this procedure poses the risk of sacrificing the normal PTT, potentially leading to iatrogenic flatfoot [12,13]. Furthermore, there is a theoretical risk of weakening the plantar flexor of the ankle, which may affect heel rise.

PTT lengthening may correct abnormal IR of the talus while preserving PTT function [11]. In addition, the laxity of the lateral ligamentous structures that occurs as VAA progresses also contributes to the abnormal IR of the talus and varus ankle deformity [9]. Therefore, if PTT lengthening is performed in conjunction with lateral ligament augmentation, the abnormal IR of the talus may be more effectively corrected. However, there is a paucity of knowledge regarding the efficacy of soft tissue procedures in conjunction with SMO for VAA.

The aim of this study was to evaluate the clinical and radiographic results of SMO combined with PTT release and lateral ligament augmentation for VAA with a large TT.

## 2. Materials and Methods

### 2.1. Subjects

This retrospective study included 15 patients (15 ankles) with VAA with a large TT (greater than 5°) who were treated by the senior author from January 2015 to September 2018. This study was approved by the Institutional Review Board of our hospital, which waived the requirement for informed consent because of its retrospective design. This study was conducted on patients who met the inclusion and exclusion criteria, as described in Table 1.

Muscle strength was assessed by a thorough physical examination and an electrophysiologic study preoperatively in all cases. Manual examination and electrophysiologic study revealed normal muscle strength in all patients. The demographic and baseline characteristics are shown in Table 2.

### 2.2. Clinical Evaluation

Outpatient follow-up was conducted at six weeks, three months, six months, and one year postoperatively, with subsequent annual follow-ups. Clinical evaluation was performed preoperatively and annually postoperatively by an independent nurse who was not involved in the surgery. Clinical results were evaluated using the visual analog scale (VAS) for pain, the American Orthopedic Foot and Ankle Society (AOFAS) ankle-hindfoot score [14], and the ankle osteoarthritis scale (AOS) preoperatively and at the last follow-up [15]. In addition, postoperative complications were assessed through a chart review.

### 2.3. Radiographic Evaluation

**Figure 1:**
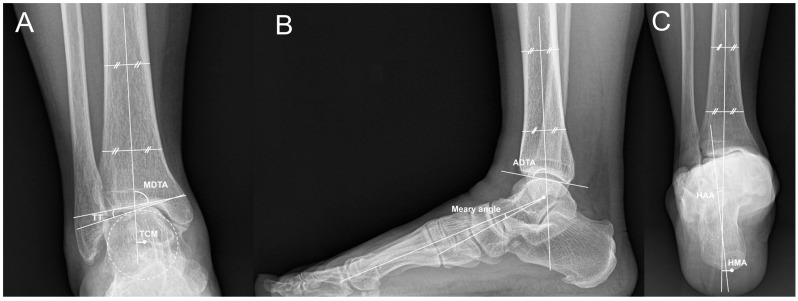
Weight-bearing ankle anteroposterior radiograph showing measurements of the medial distal tibial angle (MDTA), the talar tilt (TT), and the talus center migration (TCM) (**A**). Weight-bearing foot lateral radiograph showing measurements of the anterior distal tibial angle (ADTA) and the Meary angle (**B**). Hindfoot alignment radiograph showing measurements of the hindfoot alignment angle (HAA) and the hindfoot moment arm (HMA) (**C**).

Magnetic resonance imaging was performed in all patients preoperatively to confirm cartilage, tendons, and ligaments. Radiographic evaluation was performed preoperatively, at six weeks, three months, six months, and one year postoperatively, and annually thereafter. Radiographic evaluation was performed by independent authors who were not involved in the surgery. Radiographic results were evaluated using weight-bearing ankle anteroposterior (AP) and lateral radiographs and hindfoot alignment radiograph, and all weight-bearing radiographs were taken in double-limb standing position. All radiographs were obtained using the same facility and radiographic protocol. Medial distal tibial angle (MDTA) and anterior distal tibial angle (ADTA) were defined as the angles between the tibial long axis and the tibial plafond on AP (Figure 1A) and lateral radiographs (Figure 1B) [7]. TT was defined as the angle between the tibial plafond and talar dome on AP radiograph [7]. Talar center migration (TCM) was defined as the shortest distance between the tibial axis to the center of the talus on the weight-bearing ankle AP radiograph (Figure 1A), and a positive number indicating that the talus center is medial to the tibial axis [16]. Meary angle was defined as the angle between the line bisecting the talar neck and body and the longitudinal axis of the first metatarsal, with a positive number indicating cavus orientation (Figure 1B) [17]. Hindfoot alignment angle (HAA) was defined as the angle between the tibial and calcaneal axes, with the calcaneal axis defined as the bisecting line of the angle formed by two lines representing the lateral and medial osseous contours of the calcaneus (Figure 1C) [18]. Hindfoot moment arm (HMA) was defined as the perpendicular distance between the tibial axis and the most inferior point of calcaneus (Figure 1C) [18]. HAA and HMA were represented as positive numbers when the hindfoot was varus oriented. Radiographic stage of ankle arthritis was determined using the modified Takakura classification system [5]. The explanation of the modified Takakura classification is presented in Table 3 [5].

The reliability of all radiographic parameters was evaluated. Two foot and ankle surgeons independently measured the radiographic parameters, and the measurements were repeated three weeks later. Intra- and interobserver reliabilities for radiographic parameters were analyzed using intraclass correlation coefficients. Reliability was classified as little if any (correlation coefficient, ≤0.25), low (0.26 to 0.49), moderate (0.50 to 0.69), high (0.70 to 0.89), or very high (≥0.90) [19].

### 2.4. Surgical Technique

All surgeries were performed by one foot and ankle specialist (CHP) with more than 10 years of surgical experience in foot and ankle disease. The patient was placed in a semi-lateral position to allow for both supine and lateral positions. Surgery was performed in the following order: bone procedure, medial soft tissue lengthening, and lateral soft tissue augmentation. SMO was performed using the conventional technique described in previous reports [8]. A longitudinal incision was made medially and laterally along the distal tibia and fibula. The medial incision allowed simultaneous access to the medial ankle joint and PTTs. Through the incision, open arthroscopy was performed to confirm the condition of the cartilage and the thickened soft tissue and osteophytes of the medial and lateral gutters were removed. Deltoid ligament release was not performed in all patients due to the risk of compromising the blood supply to the talus when performed at the same time as PTT lengthening. The tibia and fibula were then osteotomized toward the proximal margin of the tibiofibular syndesmosis. A medial opening wedge tibial osteotomy was performed so that the MDTA was approximately 95°, and an autogenous iliac block bone graft was placed at the osteotomy site. Osteotomized tibia and fibula were fixed with a 3.5 mm straight bending medial distal tibial plate (Synthes, Paoli, PA, USA) and a one-third tubular plate (Synthes, Paoli, PA, USA). In the three cases where severe hindfoot varus remained after SMO, an additional lateral closing CO was performed.

**Figure 2:**
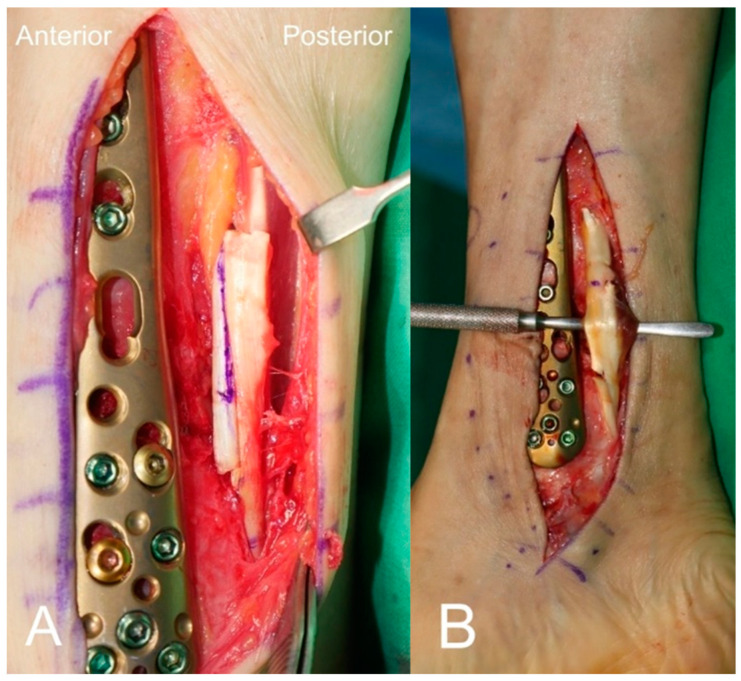
Lengthening of the posterior tibial tendon was performed using a Z-plasty (**A**) and a double hemisection (**B**).

Following completion of the bony procedures, PTT lengthening was performed. The facia covering the PTT was incised 5 cm proximal to the tip of the medial malleolus to expose the PTT. Lengthening of the PTT was performed using a Z-plasty (Figure 2A) in 13 cases and a double hemisection (Figure 2B) in two cases. The transected tendon was sutured with the ankle in neutral position and with little tension on the PTT. Finally, the modified Bröstrom operation (MBO) was performed using the conventional method reported previously [20]. The interior extensor retinaculum (IER) was identified and the joint capsule including the ATFL and CFL was detached to the lateral malleolus. Subsequently, imbrication of the lateral ligament complex was performed using two all-suture anchors (TruShot with Y-Knot^®^, Conmed, Largo, FL, USA) and reinforcement of the IER was achieved using absorbable sutures.

A short leg cast was applied in the neutral position for six weeks postoperatively, followed by an air-stirrup brace for another six weeks postoperatively. Partial weight-bearing with the brace was allowed six weeks postoperatively, and full weight-bearing was allowed 10 weeks postoperatively.

### 2.5. Statistical Analysis

All dependent variables were tested for normality of distribution and data normality was determined using the Kolmogorov–Smirnov test. To analyze the changes in the radiographic stage, stages 3A, 3B, and 4 of the modified Takakura classification were assigned quantitative scores of 3, 4, and 5, respectively. The Wilcoxon signed rank test was used to compare the pre- and postoperative results. For all tests, *p*-values < 0.05 were considered significant.

## 3. Results

The intra- and interobserver reliabilities of radiographic measurements are shown in Table 4. All radiographic parameters showed more than high intra- and interobserver reliabilities.

**Figure 3:**
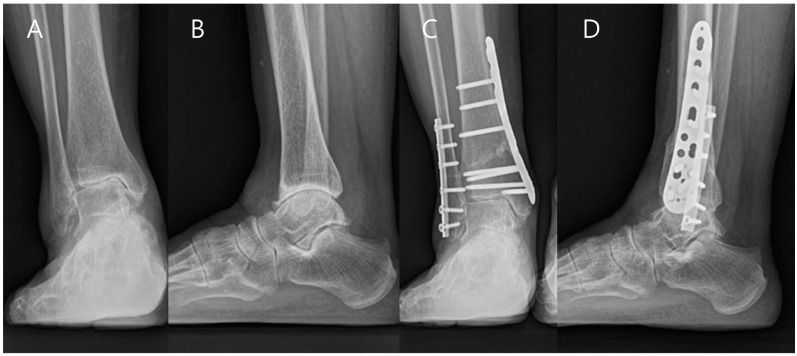
Preoperative radiographs showing stage 3B arthritis with talar tilt (**A**,**B**). Radiographs taken 2 years after supramalleolar osteotomy combined with posterior tibial tendon release showing stage 2 arthritis with improvement of talar tilt (**C**,**D**).

**Figure 4:**
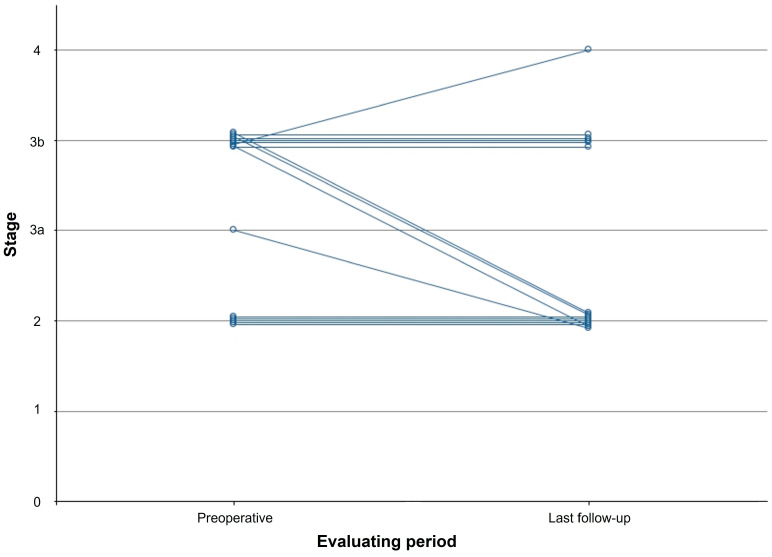
Graphs show the sequential change of radiographic stage over time.

At the last follow-up, clinical results including VAS, AOFAS ankle-hindfoot score, and AOS were significantly improved postoperatively (Table 5). All radiographic parameters changed significantly postoperatively; however, the Meary angle showed no significant change postoperatively (Figure 3). Four patients showed improvement in radiographic stage postoperatively (Figure 4); however, average radiographic stage did not significantly improve postoperatively in all patients.

Hardware was removed in all patients due to irritation of hardware. One patient with preoperative stage 3B showed progression of arthritis to end-stage postoperatively and underwent additional arthrodesis at two years postoperatively.

## 4. Discussion

In this study, we evaluated the clinical and radiographic results of SMO combined with PTT lengthening in VAA with TT. Clinical outcomes and TT showed significant improvement without complication after surgery, but no significant improvement in ankle arthritis stage was obtained. In one case, the stage worsened from preoperative stage 3B to postoperative stage 4. Various factors may have contributed to this rapid progression of arthritis. Although the MDTA and hindfoot alignment were sufficiently corrected after SMO, it is believed that there was excessive lateral migration of the talus center, which resulted in excessive lateral loading of the talus.

Most of the published literature on VAA has primarily focused on realignment of the lower extremity using bony corrections, such as SMO and CO [1,2,3,4,5,7,8]. SMO is the most commonly performed joint preserving procedure for VAA; however, there is still debate regarding the appropriate indication for SMO in VAA [5,8]. Studies have defined the appropriate indication for SMO as the degree of TT. Some studies do not recommend SMO in stage 3B arthritis with a TT of 10° or more [5], and other studies have reported unfavorable outcomes after SMO in VAA with a TT of 7.3° or more [8]. There is a study that suggests advanced ankle arthritis of stage 3B itself is a risk factor for SMO failure [21]; however, the latter study was conducted in traumatic arthritis with ankle fracture or chronic ankle instability, so it is difficult to directly relate to the study of primary VAA as in this study.

In surgical treatment of arthritis, proper soft tissue tension balancing is crucial for good long-term outcomes. The order of soft tissue tension balancing in joint surgery is typically ligament release, tendon release, and bony release [22,23]. However, the method and sequence of soft tissue release in ankle arthritis is still not well defined and is mostly based on personal experience. As ankle arthritis progresses, the medial soft tissues tighten and the lateral soft tissues loosen [24]. Therefore, it is common to lengthen the medial side and tighten the lateral side for soft tissue balancing. For lateral augmentation, MBO is commonly used [2,25,26]; however, the method of medial soft tissue release is not clearly defined. After performing SMO, talar reduction to the mortise is checked under fluoroscopy and additional medial release is considered if reduction is not achieved [24]. Talar reduction can be hindered by a medial soft tissue contracture, including the deltoid ligament and PTT. Therefore, theoretically, the first step in medial soft tissue release is release of the deltoid ligament, and if it is insufficient, release of the PTT can be added.

Recently, Park et al. reported surgical treatment using PTT transfer combined with SMO for VAA with a large TT greater than 7.5° [11], which was inspired by the method performed in paralytic VAA [27]. Studies using weight-bearing CT have shown that abnormal IR of the talus is observed in cases of VAA with a large TT, and PTT is one of the important causes of abnormal IR of the talus [9]. Therefore, PTT transfer can eliminate the force that causes abnormal IR of the talus by detaching the PTT, and create a reducing force of the talus by transferring the PTT to the cuboid bone. However, this method has the risk of causing iatrogenic flatfoot by performing PTT transfer in patients without paralysis [12,13]. In addition, since bone to tendon healing requires more time than bone union, the combination of SMO and PTT transfer results in a longer non-weight-bearing period and rehabilitation period than SMO alone. Furthermore, there is risk of postoperative infection and skin necrosis due to the large number of surgical incisions and long operation time. Therefore, to overcome these limitations, we tried to perform PTT lengthening and lateral ligament reconstruction instead of PTT transfer in TAA with TT and obtained satisfactory outcomes without complications. Compared with the results of Park et al. [11], this study did not show a significant improvement in ankle arthritis stage but showed a significant improvement in TT and clinical outcomes. However, to prove the non-inferiority of our surgical method, a randomized controlled trial comparing our surgical method with PTT transfer is needed.

In VAA, TT typically increases as arthritis progresses. However, there is still a lack of evidence regarding the exact threshold of TT at which conventional SMO can achieve correction of VAA. Lee et al. found that VAA with a TT greater than 7.5° is difficult to achieve good results with conventional SMO [8], and Park et al. also applied this criterion to define a large TT [11]. Theoretically, combined procedures of PTT lengthening and MBO are expected to have weaker correction force for abnormal IR of the talus compared to PTT transfer. Although MBO creates a static correction force for abnormal IR of the talus, we thought it would be much weaker than the dynamic correction force created by PTT transfer. Therefore, unlike previous studies, we defined a large TT as a TT greater than 5° in this study.

In the study by Park et al., when SMO was performed in combination with PTT transfer in the VAA and the outcome was compared between the improved and unimproved groups, there was a significant lack of postoperative correction of hindfoot varus in the unimproved group [11]. Although the study did not perform a logistic regression analysis of factors affecting outcome, it is believed that residual hindfoot varus may be one of the reasons for surgical failure. In the current study, we performed simultaneous lateral closing CO in three cases with severe hindfoot varus. Although hindfoot varus can be corrected by SMO and PTT lengthening, the degree of correction cannot be quantified. Therefore, we decided to perform additional CO not preoperatively, but after confirmation of residual hindfoot varus by fluoroscopy and manual examination after SMO and PTT lengthening. In this study, the preoperative HAA was 6° of varus, which was corrected to 1° of valgus postoperatively, and sufficient correction of hindfoot varus was achieved, and performing CO together was considered if necessary.

This study is the first to achieve clinical outcomes by performing PTT lengthening and lateral ligament augmentation to balance soft tissue tension and correct abnormal IR of the talus. The results of this study demonstrated the feasibility of SMO combined with PTT lengthening and lateral ligament augmentation in advanced arthritis with a large TT. However, there are several limitations in this study. First, the number of subjects included in the study is small and it is a retrospective study. There is not much evidence for PTT lengthening in VAA with a large TT, and the appropriate indication for this procedure has not yet been established. Therefore, this study is a pilot study, and a larger study with more patients will be conducted based on the results of this study. Second, this study is limited by the lack of a comparison group. A comparative study of SMO alone or SMO combined with PTT transfer is needed to prove the superiority of our technique. Therefore, a randomized controlled trial with a large number of patients is needed to find the best surgical method for VAA with a large TT.

## 5. Conclusions

In this study, clinical outcomes and TT showed significant improvement postoperatively; however, no significant improvement in ankle arthritis stage was achieved. Therefore, PTT lengthening and lateral ligament augmentation combined with bony realignment procedures may be a reasonable option for treating VAA with a large TT greater that 5°.

## Figures and Tables

**Table 1:** Inclusion and exclusion criteria.

**Inclusion Criteria**
Adult patients (>18 years) who failed to respond to ≥3 months of other conservative treatments
Primary ankle osteoarthritis
Varus ankle osteoarthritis
Patients with a follow-up period of >12 months
**Exclusion Criteria**
Osteoarthritis with flatfoot or hindfoot valgus
Valgus ankle osteoarthritis
Traumatic ankle osteoarthritis due to fracture around ankle
Paralytic ankle arthritis with muscle weakness

**Table 2:** Demographics of patients.

	N (%)	Mean (Range)
Age (years)		64 (53–75)
Sex (male/female)	9 (60)/6	
Body mass index (kg/m^2^)		26.4 (23.1–30.9)
Follow-up periods (months)		19.4 (12–50)
Smoker	2 (13.3)	
Diabetes	3 (20)	
Modified Takakura classification		
2	5	
3A	1	
3B	9 (60)	

**Table 3:** Modified Takakura classification.

1	No narrowing of the joint space, but early sclerosis and formation of osteophytes
2	Narrowing of the medial joint space
3	Obliteration of the medial joint space with subchondral bone contact
3A	Obliteration of the joint space was limited to the medial malleolus
3B	Obliteration extended to the roof of the dome of the talus
4	Obliteration of the whole joint space with complete bone contact

**Table 4:** Intra- and interobserver reliabilities of radiographic measurements.

Parameters	Interobserver Reliability (95% CI)	Intraobserver Reliability (95% CI)
Medial distal tibial angle	0.95 (0.83–0.98)	0.93 (0.88–0.97)
Anterior distal tibial angle	0.97 (0.91–0.99)	0.92 (0.82–0.96)
Talar tilt	0.91 (0.80–0.95)	0.88 (0.75–0.94)
Talus center migration	0.84 (0.72–0.90)	0.81 (0.69–0.88)
Meary angle	0.91 (0.81–0.97)	0.83 (0.70–0.91)
Hindfoot alignment angle	0.87 (0.72–0.96)	0.81 (0.71–0.91)
Hindfoot moment arm	0.92 (0.88–0.98)	0.90 (0.86–0.95)

CI, confidence interval.

**Table 5:** Pre- and postoperative clinical and radiographic results.

Parameters	Preoperative	Last Follow-Up	*p*-Value
VAS	7.5 ± 0.9	3.1 ± 2.4	<0.001
AOFAS score	54.4 ± 8.7	82.5 ± 17.9	<0.001
AOS	72.6 ± 11.5	34.5 ± 23.3	<0.001
MDTA (°)	86.8 ± 3.3	94.3 ± 2.9	<0.001
ADTA (°)	78.8 ± 2.4	80.9 ± 2.7	0.002
TT (°)	6.9 ± 1.7	3.8 ± 2.3	0.001
TCM	3.5 ± 1.6	−0.1 ± 1.1	<0.001
Meary angle (°)	2.8 ± 5.3	0.7 ± 4.7	0.038
HAA (°)	6.9 ± 3.5	−0.6 ± 4.6	<0.001
HMA	7.5 ± 5.6	−5.6 ± 6.5	<0.001
Takakura stage *	3.3 ± 1.0	2.9 ± 1.1	0.111

Values are mean ± SD. VAS, visual analog scale; AOFAS, American Orthopaedic Foot and Ankle Society; AOS, ankle osteoarthritis scale; MDTA, medial distal tibial angle; ADTA, anterior distal tibial angle; TCM, talus center migration; TT, talar tilt; HAA, hindfoot alignment angle; HMA, hindfoot moment arm. * In order to analyze the changes in radiographic stage, stages 3A, 3B, and 4 of the modified Takakura classification were assigned quantitative scores of 3, 4, and 5, respectively.

## Data Availability

Data are not available due to restrictions of privacy.
